# Magnetorheological Elastomers: Fabrication, Characteristics, and Applications

**DOI:** 10.3390/ma13204597

**Published:** 2020-10-15

**Authors:** Sung Soon Kang, Kisuk Choi, Jae-Do Nam, Hyoung Jin Choi

**Affiliations:** 1 Department of Polymer Science and Engineering, Inha University, Incheon 22212, Korea; 22191263@inha.edu; 2 Department of Polymer Science and Engineering, School of Chemical Engineering, Sungkyunkwan University, Suwon 16419, Korea; kisuk929@skku.edu (K.C.); jdnam@skku.edu (J.-D.N.)

**Keywords:** magnetorheological, elastomer, magnetic particle, viscoelastic, rheological

## Abstract

Magnetorheological (MR) elastomers become one of the most powerful smart and advanced materials that can be tuned reversibly, finely, and quickly in terms of their mechanical and viscoelastic properties by an input magnetic field. They are composite materials in which magnetizable particles are dispersed in solid base elastomers. Their distinctive behaviors are relying on the type and size of dispersed magnetic particles, the type of elastomer matrix, and the type of non-magnetic fillers such as plasticizer, carbon black, and crosslink agent. With these controllable characteristics, they can be applied to various applications such as vibration absorber, isolator, magnetoresistor, and electromagnetic wave absorption. This review provides a summary of the fabrication, properties, and applications of MR elastomers made of various elastomeric materials.

## 1. Introduction

As the increasing trend of better and comfortable lifestyle has led to growing demand for both new technologies and materials, advanced smart and intelligent functional materials have been receiving a large attentions in recent years [1]. The smart materials are those controllable with external environments such as electric or magnetic field, mechanical stress, heat, and light [2].

Among these, magnetorheological (MR) materials become one of the most important smart materials in terms of their huge industrial potentials. They are classified as a functional smart material possessing tunable rheological and viscoelastic properties such as yield stress, shear stress, dynamic moduli, and damping property when an external magnetic field is applied [3,4,5,6,7]. Note that while their electrical analogue electrorheological (ER) materials have been also extensively investigated [8], MR materials prevail ER materials regarding both their scientific investigation and applications due to superior performance characteristics of the MR materials [9].

MR materials in general include several different systems, depending on their media employed such as MR fluid (MRF), MR foams, MR gels, and MR elastomer (MRE) [4]. Historically, MRFs, colloidal suspensions that consist of small soft-magnetic particles with extremely small hysteresis, high magnetic permeability and high saturation magnetization suspended in non-magnetic liquids [10] have been widely studied. Dispersed soft-magnetic particles enhance the apparent shear viscosity and yield stress of the MR suspensions [11], depending on both magnetic field strength applied [12] and concentration of micron-sized magnetic particles [13,14]. They have been extensively adopted in various applications such as MR brake, MR valve, MR mount, MR clutch, and MR damper [15]. The versatility of their wide applications is caused by noiseless operation, rapid field responsiveness of MRFs relative insensitivity to small quantities of contaminants or dust, and easy control [16]. On the other hand, despite that MRFs are possessing significant merits with many potential applications, their disadvantages include sealing issues due to the leakage of the medium liquid, contaminating environment, and sedimentation of the particles [17]. Hence these drawbacks limit to the further expansion of its engineering applications.

Contrary to the MRFs, MREs are the hard (particles) and soft (pristine matrix) hybrid composite smart elastomeric materials [18]. Therefore, unlike MRFs, solid-state MREs overcome the disadvantages of MRFs. MRE contains unique mechanical properties in such a way that their viscoelastic properties change under an applied magnetic field. This behavior is very similar to the MRF, but MRE is a more like solid equivalent [19]. In addition, the MRE is generally composed of a material that contains a rubber matrix, minor additives and magnetic carbonyl iron (CI) particles. In such a material, the coalescence of CI particles occurs within an applied magnetic field, which attributes to the hardness, enhanced elastic modulus, and shear modulus [20]. The typical rubber matrix includes silicone rubber (SR) [21,22,23], polybutadiene rubber [24,25], nitrile rubber [26], and polyurethane (PU) rubber [27,28] and others. Traditionally, elastomers are nonmetallic materials, being used in wide engineering applications such as seals, gaskets, and tires [29]. Correspondingly, the MREs are mainly used in three main categories in past decades, which are sensing devices, actuators and vibration, and noise control [30]. In recent studies, the MRE is also adapted in biomedical engineering field where it is used as a soft material for magnetic-elastic soft robot [31,32]. The tunable viscoelastic properties of MREs have also overpowered the mobility limitation of small-scale robot due to texture or different material in an unstructured environment [31]. Hence, according to these studies, MREs have drawn dramatic interests in various engineering applications.

On the other hand, MREs can be classified into two different groups, which are isotropic and anisotropic MREs, based on mechanisms of magnetically polarized particle configuration in the MREs. The polarized particles are uniformly suspended in an isotropic MRE, so that the MRE shows homogenous physical behaviors in every direction. For an anisotropic MRE, the magnetic particles are aligned along with the input magnetic field direction during manufacturing process, which leads to the perpendicular direction of a flat MRE specimen. In many studies, the shear direction that is perpendicular to the CI particles’ alignment was observed when a rheological measurement of anisotropic MRE was conducted [33,34,35]. Tian and Nakano [36] proposed the fabrication process of anisotropic MRE with 45° CI particles’ (less than 60 µm depending on their shape) arrangement in various silicone oil concentrations, which enhanced the storage modulus (G’). In addition, the combination of isotropic/anisotropic MRE has examined to adjust damping capability and zero-field dynamic stiffness of silicone based MRE [37]. According to these studies, MRE has raised great attention in engineering fields. On the other hand, along with the CI particles, various magnetic particles such as CoFe_2_O_4_ (less than 12 nm) [38], Ni (10 µm) [39], FeCO_3_ (less than 30 nm) [40], and industrial waste nickel zinc ferrite (less than 2 µm) [41] have been also employed. Siti et al. [42] fabricated MREs using CI and nano-sized Ni-Mg cobalt ferrites (35 to 80 nm) based on SR. Even though, both MRF and MRE possess similar magnetic field properties, the main distinction is that their operation period is dependent within two types of materials. The interesting point is that MRE tends to operate in pre-yield regime [43,44], where MRF is typically operated in a flow regime or post-yield shear regime [45,46]. Hence, the magnetic field performance of MRF is mainly obtained from yield stress while MRE is characterized by field-dependent modulus [47].

This article reviews a recent trend on preparation, characteristics, and applications of smart MRE materials. While the microstructures with isotropic or anisotropic MREs are tested by scanning electron microscopy (SEM) and the magnetic property of the magnetic particles used was analyzed by VSM, the mechanical characteristics of MREs such as tensile, Payne, and loss factor have been conducted. In addition, the rheological properties of MREs including dynamic, amplitude sweep, frequency, sweep, and creep test are also illustrated.

## 2. MRE Materials

### 2.1. Fabrication

**Figure 1:**
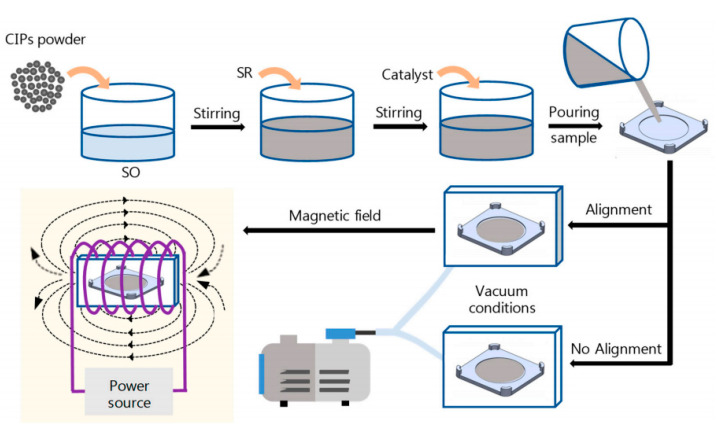
Schematics of manufacturing step of both isotropic and anisotropic magnetorheological elastomer (MREs) (stirring, mixing, and alignment with or without magnetic field) [48].

The MRE is generally treated as solid analogues of MRF, being consisted of magnetized particles in polymer medium. The manufacturing procedure of MRE is similar to that of the ordinary rubbers. The materials include soft-magnetic particles, elastomers, and additives. The MREs can be categorized into two classes, which are isotropic and anisotropic upon the curing condition whether it requires applied magnetic field or not. The fabrication process of MRE includes three steps: Mixing, curing, and magnetic particle alignment under an input magnetic field as presented in Figure 1. The isotropic MRE is without a magnetic field while an anisotropic MRE requires an applied magnetic field. For example, Puente-Córdova et al. [48] prepared MREs with two different concentration (20 and 30 wt%) of CI, silicone oil, and tin catalyst. The magnetic particles were dispersed in silicone oil and mixed with SR and catalyst at room temperature. For such high viscosity of the solution requires high mixing process to minimize the sedimentation of the added magnetic particles. The curing process is usually conducted within 12 h under vacuum environment to avoid porosity. The homogenous mixture was exposed to a magnetic flux density of 7 mT only for anisotropic reinforced samples. It is crucial to note that the mixture reaches the semi-solid state, which mitigate magnetic particle sedimentation after 20 min of curing process.

### 2.2. Soft-Magnetic Particles

MREs are fabricated by adding high magnetic permeability particles to a viscoelastic material [49]. As such, the magnetic particles added to the MR elastomer play an important role in the MR effect. In general, the conditions of good magnetic particle for MR materials require high saturation magnetization, low remnant magnetization, and high, short-term inter-particle attraction. Of the many magnetically permeable particles, the CI which was discovered by BASF in 1925 produced by thermal decomposition of iron pentacarbonyl (Fe(CO)_5_) is the most studied and used as MR particle [50,51,52,53]. The reason is that as one of the soft-magnetics, it has the advantage of high saturation magnetization, easy magnetization and demagnetization, and no magnetic hysteresis. Because of these properties, it has been used in many fields such as MRFs [54], MR foams [55], and MR greases [56]. CI particles are soft magnetic microparticles being categorized in two groups based on their shape, which are spherical and flake-typed with a size of 1–7 µm and 5–50 µm, respectively [50,54]. In the case of MRF, due to the sedimentation problem caused by high density difference between medium oil and CI particles, their sedimentation problem has been improved by making their density relatively low by being coated with several organic materials. For example, coating polymers such as poly(methyl methacrylate) (PMMA) [6], PS [7], polyaniline [44], and polycarbonate [57] not only reduce the density of CI particles but also prevent chemical oxidation of CI surfaces. However, unlike MRFs, particle sedimentation is not a problem for MREs. The problem with MREs is the compatibility between the magnetic particles and the elastomeric medium. The reason is that CI particles are hydrophilic, whereas elastomer matrices are generally hydrophobic. Therefore, many studies have been conducted to increase the surface compatibility of the CI matrix with the elastomer matrix.

**Figure 2:**
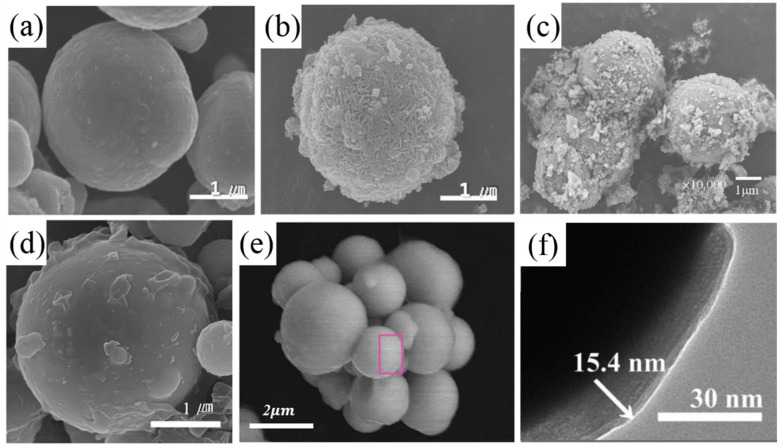
SEM image of (**a**) pure carbonyl iron (CI), (**b**) CI/(3-aminopropyl) triethoxysilane (APTES) [58], (**c**) CI/poly(methyl methacrylate) (PMMA) [59], (**d**) CI/poly(glycidyl methacrylate) (PGMA) [60], (**e**) CI/poly(fluorostyrene) [61], (**f**) CI/poly(trimethylsilyloxyethyl methacrylate) (PHEMATMS) [62].

Among various surface modification methods, the treatment of CI particles with silane coupling chemicals is known to be a very economical and efficient way to increase the affinity between the CI particles and the medium [58]. An et al. [58] studied (3-aminopropyl) triethoxysilane (APTES) modified CI particles to improve compatibility with the elastomer matrix (Figure 2b). They fabricated a natural rubber based anisotropic MRE using CI particles surface modified with APTES. And they measured the properties of MRE according to the surface treatment. In order to coat the CI surface with APTES, hydrochloric acid was used for the pre-treatment. This enables the CI surface to be coated by activating the OH group to polymerize APTES onto the CI surface. Figure 2b is a SEM image, showing that the surface of the CI has a rough surface by APTES coating. When CI is coated with APTES in this way, it has NH_2_ group on the surface of CI, making CI particles to have a good affinity with natural rubber.

Many studies have been carried out on coating CI particles with PMMA and applying them to MRFs [6,63]. The PMMA-coated core–shell structured CI particles have a lower density than neat CI particles, resulting in better dispersion stability in the fluid. Li et al. [59] have applied CI/PMMA core–shell particles to MREs rather than MRFs (Figure 2c). The rough surface represents how the CI particles were covered by PMMA particle. Fabricating MREs with CI/PMMA core–shell particle results in lower Payne effects by increasing bond strength between matrix and particles. CI particles were coated with PMMA by emulsion polymerization. First, the surface of the CI particles was activated with acetic acid and then dispersed in distilled water. Sodium lauryl sulfonate is used as a stabilizer to suspend the CI particles, and the CI surface is coated with PMMA using MMA as a monomer and ammonium persulfate as an initiator. As shown in the Figure 2c, PMMA coated CI surface can be confirmed. Using the MRE made of CI/PMMA core–shell particles, the compatibility between particle and matrix is increased and the relative motion between particle and medium is reduced, resulting in smaller and more stable loss factors.

Kwon et al. [60] coated poly(glycidyl methacrylate) (PGMA) on CI particles and applied them to MREs and studied their properties (Figure 2d). The surface of the CI/PGMA particles was observed to be rougher than those of the pristine CI particles. The process of producing core–shell particles using the dispersion polymerization method has the advantage of being simple in a single step process. Due to the hydrophobicity of PGMA, coating PGMA on magnetic particles has the advantage of increasing chemical affinity between the CI particles and the elastomer. PGMA coated CI particles increase the hardness of the MRE matrix, resulting in lower MR effects than MREs made from pure CI. PGMA coatings enhance the bond strength between the CI particles and the elastomeric medium, reducing the loss tangent.

Fuchs et al. [61] researched the coating of poly(fluorostyrene) on CI particles with the ATRP method and applied them to MREs (Figure 2e). By coating poly(fluorostyrene) on the CI surface, it was possible to prevent the oxidation of magnetic particles, one of the potential problems with MRE. It prevents mechanical property from decreasing due to the rapid oxidation of magnetic particles, making it possible to apply for a longer time as a vibration isolator.

Martin et al. [62] reported the characteristics of MREs by coating poly(trimethylsilyloxyethyl methacrylate) (PHEMATMS) on the surface of CI particles, in which the CI-g-PHEMATMS particles showed about 6.2% reduction in magnetization than neat CI particles due to the 10–20 nm coating thickness. However, it was possible to improve thermo-oxidation stability and anti-acid/corrosion properties through polymer coating. In the case of neat CI, wettability is low, which brings a strong reinforcing effect to the MRE. However, when CI is coated with PHEMATMS, mobility is improved inside the elastomer matrix, creating a plasticizing effect. As a result, MRE with CI-g-PHEMATMS particles shows lower G’ and higher G” than MRE with neat CI. This results in a higher damping factor. In addition, the increase in particle mobility increases the relative MR effect, which is expected to be a more practical application.

In addition to CI particles, the effects of many types of magnetic particles such as CoFe_2_O_4_ [38], Ni [39], FeCO_3_ [40], and manganese zinc ferrite [41] have been studied. Siti et al. [42] fabricated MREs using CI and nano-sized Ni-Mg cobalt ferrites based on SR, and studied various properties including field-dependent viscoelastic characteristics of MREs. The experimental results showed that the G’ and loss factor were increased in MRE containing Ni-Mg cobalt ferrite nanoparticles. This means that Ni-Mg cobalt ferrite nanoparticles increase the interaction between MRE and particles. On the other hand, Nordalila et al. [64] manufactured MRE using industrial waste nickel-zinc ferrite. Experiments were conducted to compare the degree of swelling according to the content of nickel-zinc ferrite. The thermal analysis of MRE and the MR effect of anisotropic and isotropic MREs were also performed.

Recently, in addition to these soft magnetic particles, studies have been made on MREs using hard magnetic fillers [21,65,66,67]. Conventional soft magnetic particle-based MREs aimed at maximizing the MR effect. But the MR elastomer fabricated with hard magnet behaves like a flexible permanent magnet. Due to these characteristics, hard magnet-based elastomers have been applied to other applications. When an input magnetic field is present, hard magnetic particles cause rotational motion, which can cause motion and force like a magnetic field-controlled actuator. Koo et al. [21] studied the actuation properties of hard magnet MRE (H-MRE) using barium hexaferrite, strontium ferrite, samarium cobalt, and neodymium magnet. It offers the possibility that MREs with a hard magnet base can be used as magnetically controlled actuators. Therefore, it can be noted that MREs were studied by applying various magnetic particles from soft magnetic to a hard magnet.

### 2.3. Types of Elastomer

#### 2.3.1. Silicone Rubber

Unlike other organic rubbers, the silicone rubber (SR) has both organic and inorganic properties due to Si-O bonding. Because of these characteristics, heat resistance, its electrical insulation, abrasion resistance, and chemical stability are superior to general organic rubber [24,26]. Therefore, it has been used in many industrial areas including food processing and medical devices. Because of these advantages, it has also been used as a matrix of MREs. Among many SRs, polydimethylsiloxanes (PDMSs) were adopted as the matrix of MRE. Due to the chemical neutrality and stability of PDMS system, it has also a good adhesive property with metal. PDMS rubbers have the advantage of being able to cure at low temperatures and fast curing at high temperatures. In addition, the precursor has a low shear viscosity, making it easy to manufacture MREs [27,68,69]. Xu et al. [70] manufactured a PDMS based MRE and studied the movement mechanism of magnetic particles in the MRE according to the magnetic field. Li and Nakano [71] have reported the mechanical and MR effects of the CI and PDMS matrix base MRE.

#### 2.3.2. Natural Rubber

Natural rubber (NR) is a polymer of isoprene made by solidifying latex obtained from the Hevea brasiliensis tree, being generally composed of cis-constituted C_5_H_8_ units (isoprene) with one double bond in each repeat unit. NRs as unsaturated elastomers have superior characteristics such as high strength, better resistance, and elongation at break [72]. However, due to the presence of double bonds in the chain, they are very sensitive to heat and oxidation. The improvement in rubber elasticity and strength is usually obtained by a vulcanization process in the presence of sulfur, accelerators and other compounding components, creating a three-dimensional network. NR, which has undergone through this vulcanization process, has higher mechanical performance than other rubbers, making it more suitable for practical applications [30]. Chen et al. [73] used NR to study the MRE properties according to temperature, plasticizer, and iron particle content. They observed that NR had better mechanical properties than SR, and as CI contents increased, the shear modulus of MRE increased, resulting in an increase in MRE performance. However, as CI contents increased, tensile strength and angle tear strength of MRE decreased. Aziz et al. [74] studied the effects of dispersing agents such as naphthenic oil, light mineral oil, and epoxidized palm oil on MR rubber based on NR. Compared to conventional petroleum-based dispersing aids, these eco-friendly dispersing aids, also increased mechanical properties such as MRE’s magnetic behaviors, tensile strength, and elongation at break. This is because EPO increased the crosslink density of the NR matrix as dispersing aids.

#### 2.3.3. Polyurethane Elastomer

Polyurethane (PU) elastomers have a great deal of structure and properties because they can be synthesized with a wide variety of materials. For this reason, it has been used in many applications such as shoes, tires, construction, sports, electricity, etc. For the synthesis of PU elastomers, diisocyanate such as aromatic or aliphatic, macrodiol such as polymeric diol, and small molecule diol or diamine as chain extenders are used to form a copolymer. Macrodiol sequence is a soft-segment and diisocyanate and chain extender sequence are a hard-segment to have the character of elastomer [75,76,77,78]. In order to provide mechanical properties such as the elastomer of the PU, the soft segment generally has a glass transition temperature substantially lower than the desired service temperature, and the hard segment has a glass transition temperature or melting temperature much higher than the desired service temperature.

These characteristics of the PU elastomer attract much attention as the matrix of MRE. This is because properties such as tensile strength, stiffness, chemical resistance and friction coefficient of the PU elastomer matrix can be controlled well by changing the type of polyol and diisocyanate according to the application of MRE. Wu et al. [79] manufactured isotropic MREs in an in-situ one-step polycondensation procedure using CI as magnetic particles in the PU base. They improved the phase separation by using surface milling and ball milling of CI particles to increase the dispersibility of CI in the PU elastomer. On the other hand, Hu et al. [80] studied MREs with PU/Si-rubber hybrids and found that they had higher MR effects than pure Si-rubber or PU matrices.

#### 2.3.4. Ethylene/Acrylic Elastomer

Ethylene/acrylic elastomer (AEM) is known to have good low temperature properties, good resistance to compression settings, and combine the deterioration effects of heat, oil, and weather, making it be used in the automobile industries as seal and gasket [81].

Recently, Gao et al. [82] adopted the AEM for an elastomeric matrix of CI-based MRE, also using calcium carbonate as a compatibilizer to study its effects. Due to the characteristics of the positive zeta potential of calcium carbonate, it was suitable as a compatibilizer for the AEM-based MRE. As a result, the addition of calcium carbonate can improve the mechanical strength of MRE as well as the interfacial interaction of CI particles and AEM matrix along with the study of effect of different CI contents on the MRE with AEM matrix. In addition, the interfacial reaction between the CI particles and the matrix was also controlled by different manufacturing methods [83].

Relatively low Mooney viscosity of the AEM is expected such that the CI particles are easy to be dispersed better in the matrix compared to higher Mooney viscosity elastomers such as NR. The MR effect and mechanical properties were compared according to the difference between the content of CI particles and the interfacial reaction with the matrix.

#### 2.3.5. Waste Tire Rubber

Focusing on the environmental and recycling issue, Ubaidillahs et al. [84] reported waste tire rubber based MRE and studied viscoelastic properties of MRE, in which fully vulcanized scrap tire rubber was introduced as the new matrix for the MRE. The successful processing of the scrap tire rubber was undertaken using a high-temperature high-pressure (HTHP) sintering process, converting the inert scrap tires into recycled rubber. They fabricated MRE by running the HPHT sintering process at 25 MPa and 200 °C for an hour. They presented the possibility of making waste tires into highly valued recycled products through the manufacture of MRE from waste material.

#### 2.3.6. Ethylene-Propylene-Diene Monomer Rubber

Ethylene propylene diene monomer (EPDM) rubber is a type of synthetic rubber used in many applications, being widely adopted in the manufacture of cables, hoses, car tire sidewalls, cover strips, wires, belts, shoes and sporting goods, because it possesses good resistance to oxidation, aging and temperature. Additionally, EPDM rubber is easier to handle and cheaper compared to NR. Conversely, EPDM rubber has several disadvantages compared to NR, such as lower tensile strength and fatigue strength. Therefore, carbon black is used a lot as reinforcement for EPDM rubber [85].

Burgaz et al. [86] researched the effects of magnetic particles and carbon black on EPDM rubber based MRE, also focusing on their difference by making MREs with two different micron-sized iron particles of CI powder and bare iron (BI) powder. Through the analysis of Payne effect, they compared the results that CI particles are found to be more compatible with EPDM rubber matrix than BI particles, based on mechanical properties and MR properties according to the contents of CI particles in EPDM rubber based MRE.

Additionally, Plachy et al. [87] fabricated MRE by dispersing CI in EPDM rubber matrix. The degree of porous MRE was controlled through the foaming agent based on azodicarbonamide. Through this, the effect of the porous matrix on the EPDM rubber based MRE was studied. As a result, the porous system complements the high toughness of conventional MREs, which can potentially be utilized in other applications with properties between MR fluids and solid elastomers.

#### 2.3.7. Thermoplastic Elastomer

While most MREs are based on the chemically cross-linked elastomeric matrices, rather easily processable thermoplastic elastomers (TPEs) have been introduced. As desirable alternatives to typical vulcanized rubbers, they can be processed at elevated temperatures as conventional thermoplastics while maintaining their high elasticity. Lu et al. [88] adopted poly(styrene-ethylene-butylene-styrene) (SEBS) triblock copolymer as a TPE to fabricated CI based SEBS MRE and found that the isotropic composite prepared exhibited a larger storage modulus compared to the SEBS matrix at room temperature where the EB phase therein was rubbery while the PS phase was in the glassy state. In contrast, the SEBS composite prepared under the magnetic field at high T contained a chain structure of CI particles. On the other hand, improved thermal stability and mechanical strength of SEBS matrix, not sacrificing the elasticity and toughness was also observed [89].

Furthermore, as a potential injection-molding process for the MRE, polyolefin-based TPEs are also used [90,91]. Cvek et al. [90] fabricated MRE based on propylene-based TPE. Despite decreased MR effect of the MREs by tens of percent per the processing cycle, they expect that the injection-molded TPE-based MREs could offer a new pathway for producing the smart engineering composites owing to the ability to be easily reprocessed.

### 2.4. Non-Magnetic Fillers

Researches have been conducted to enhance the mechanical and MR characteristics of MREs by adding non-magnetic fillers as additives to MREs. Different types of non-magnetic fillers include reinforcing agents [92,93,94,95], plasticizers [96,97,98], and crosslink agents [99,100] that are related to the mechanical characteristics of MREs, in addition to the important magnetic fillers to increase MR properties. Mechanical reinforcing agents include carbon nanotube (CNT) [92], graphene [93], graphite [94], and carbon black [95]. Li et al. [101] fabricated MRE by adding multi-walled CNTs. The addition of a small amount of CNTs can effectively increase the mechanical properties of MREs. In addition to increasing dynamic stiffness and damping without an applied magnetic field, it also showed a higher field-induced increment. Chen et al. [102] fabricated MREs with different carbon black content and observed the microstructure and mechanical performance of the prepared MREs. As the carbon black is added, the MR effect, damping ratio, and tensile strength of the MRE are all increased.

On the other hand, plasticizers are the most common additive in rubbery materials. Generally, plasticizers are added to the mixing and vulcanization processes of elastomers to increase the flexibility, distensibility, and workability of elastomers. The addition of plasticizer to the MRE helps the magnetic particles to be arranged by the magnetic field strength during the curing of the elastomer, thus increasing the absolute MR effect. Khairi et al. [97] examined the addition of silicone oil as a plasticizer to the SR based MRE. It resulted in an increase of particle alignment and the MR effect. The addition of silicone oil generally lowers the viscosity of the uncured rubber, increasing the alignment of the magnetic particles. Kimura et al. [98] analyzed the properties of MREs by adding a plasticizer such as dioctyl phthalate, dimethyl phthalate, butyl benzyl phthalate and bis(2-methyl octyl) phthalate. As the plasticizer content increases, the G’ at 0 mT decreases and the G’ at 500 mT increases. This is because the mobility of magnetic particles of MRE is increased by the addition of plasticizer. Fan et al. [103] varied the sulfur content to examine the effect of the crosslink density of the MRE on the damping behaviors. As the crosslinking density of MRE decreases, the movement of magnetic particles increases, and the damping property increases. This confirms that the rearrangement of magnetic particles plays an important role in improving magnetic field-induced change of loss factor, G’, and relative MR performance.

While most additives for MREs are normally considered as non-magnetic, recently, addition of magnetic filler additives to the typical CI-based MRE composites has been reported to show a significant effect on the MR properties. Magnetic nano-sized fillers include both soft-magnetic and hard-magnetic nanoparticles. Lee et al. [104] prepared MRE by using micron-sized CI as a main magnetic particle in the NR matrix and adding nano-sized gamma-ferrite as an additive. The gamma-ferrite is a rod-shaped hard magnetic particle, which has been confirmed to be more uniformly aligned with the CI particles of the elastomer added gamma-ferrite than the neat CI-base elastomer due to the morphological characteristics. In addition, strain sweep tests confirmed that MREs with gamma-ferrite added had higher modulus. Kramarenko et al. [105] fabricated MRE by using SR as matrix and CI and NdFeB, a hard-magnetic filler particle, in various sizes and concentrations. MR properties and viscoelastic behavior were measured according to NdFeB size and concentration. The addition of hard magnetic NdFeB caused the non-linear viscoelastic behavior in small strains and caused a large increase in modulus.

## 3. Characteristics

### 3.1. Morphological Property

**Figure 3:**
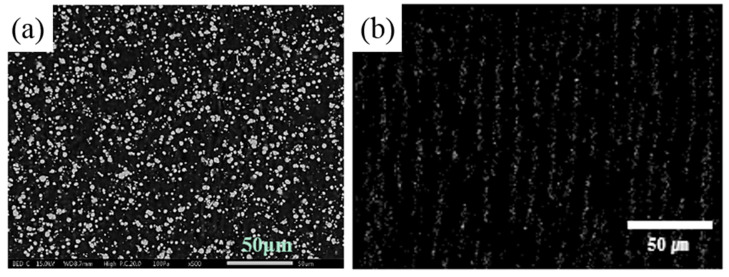
SEM images of the pure CI particles based isotropic MRE (**a**) [97] and anisotropic (**b**) MRE [104].

In the MRE, the suspension of magnetic particles and the adhesion between the CI particles and the medium are very important to the properties of the MRE. Morphology of the isotropic and anisotropic MREs is observed through scanning electron microscope (SEM) [97,104]. To observe the morphology of the MRE, the sample is soaked in liquid nitrogen and cut perpendicularly. Through this morphology observation, the adhesion between magnetic particles and matrix and dispersion state of magnetic particles can be observed. Figure 3a is an isotropic MRE prepared without a magnetic field and Figure 3b is SEM image mapped by EDX of anisotropic MRE prepared under magnetic field conditions during curing. Figure 3a shows randomly dispersed carbonyl iron particles in SR. Figure 3b is SEM images of anisotropic MRE sample. Unlike Figure 3a image, the CI particles are aligned in the elastomeric medium, and this confirms that it is anisotropic MREs. These morphological characteristics are deeply associated to the mechanical and MR characteristics of MREs and are important factors for applications.

### 3.2. Mechanical Characteristics

#### 3.2.1. Tensile Property

In general, as the amount of magnetic particles in the MRE increases, the MR effect increases but the mechanical property decreases [106,107]. The deterioration of these mechanical characteristics is a very important factor in the industrial application of MREs. For this reason, the measurement of the mechanical characteristics of MREs is very important. A good way to measure the mechanical properties of MREs is to measure their tensile strength. Wu et al. [79] conducted a study comparing the properties of surface treated CI and untreated CI on PU-based MREs. Table 1 represents the mechanical characteristics of PU and PU-CI composites with 50%, 60%, and 70% of raw and treated CI. As shown in Table 1, both the tensile strength and the elongation at break decreased with increased CI content. However, when the CI content is 70%, the comparison shows that the MRE made of the surface-treated carbonyl iron has higher tensile strength and elongation at break than the MRE made of the untreated CI. This shows that the surface treatment of CI increases the affinity of the matrix and magnetic particles, resulting in higher mechanical properties.

#### 3.2.2. Payne Effect

**Figure 4:**
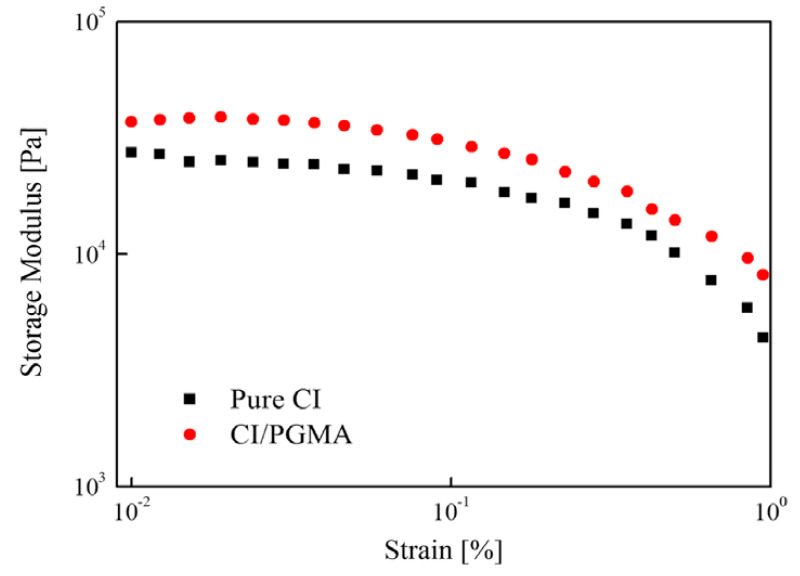
The storage moduli CI/PGMA and pure CI based magnetorheological (MR) elastomers [60].

Payne [108] reported that the effects of changes due to deformation of the microstructure of the material were due to breakage and reform of weak physical bonds connecting neighboring filler clusters. This analysis has been applied for the elastomeric composites where the filler particles are connected to the elastomer in the microstructural viewpoint. As the strain amplitude increases, the distance between the CI particles increases and the bond between the elastomer and the particle breaks. For this reason, G’ decreases as the structure breaks. In other words, if the bond strength of particles and matrix is strong, the change of G’ will be small due to the change of strain amplitude. If the bond strength of particles and elastomer is weak, the change of G’ will be large due to strain amplitude. In other words, the Payne effect represents the bond strength between the elastomeric medium and the filler particles. Many researchers have evaluated the properties of MREs through the Payne effect of anisotropic and isotropic MREs [109,110,111,112]. The Payne effect is measured by the change of G’ according to the change of strain amplitude under cyclic loading conditions and calculated using Equation (1),

(1) $$\mathrm{Payne}\;\mathrm{Effect}=\frac{G_{0}^{\prime }-G_{\infty }^{\prime }}{G_{0}^{\prime }}\times 100$$

where $G_{0}^{\prime }$ is the G’ at an initial strain and $G_{\infty }^{\prime }$ is the G’ at the infinite strain. Figure 4 is a graph showing the G’ of both pure CI based MRE and PGMA coated CI-based MRE as a function of strain [60]. The Payne effect of the CI-based MRE was calculated to be 84%, while that of PGMA coated CI-based MRE was 78%. MRE with PGMA coated CI had a lower Payne effect. This confirms that CI coated with PGMA has a stronger bond strength with the rubber matrix than the CI-based MRE.

#### 3.2.3. Damping Loss Factor

**Figure 5:**
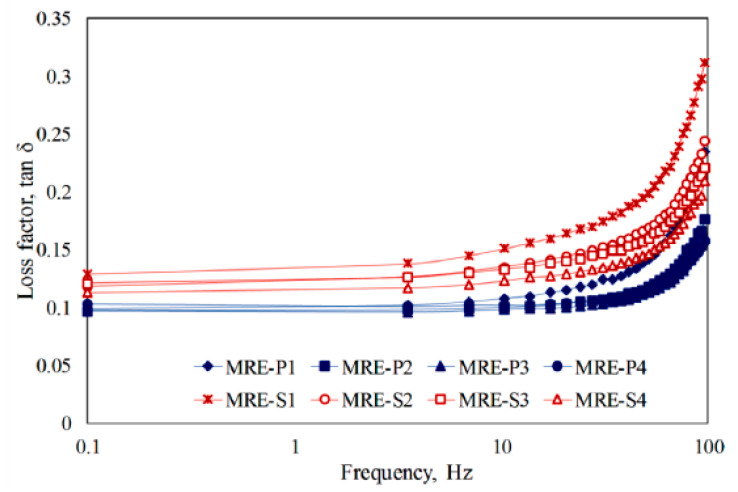
Frequency dependence of loss factor with different curing magnetic fields and particle shapes [115].

The loss factor which is also called damping factor, tan δ = G”/G’, represents the ratio of G’ to loss modulus (G”) and the degree of energy dissipation during the deformation of the material. It is adopted to measure the damping efficiency of MREs [113,114]. Dissipation occurs mainly at the interface between the matrix and the particles, that is, higher interfacial adhesion results in loss factor reduction. Hapipi et al. [115] examined plate-like CI-based MREs (MRE-P) and spherical type CI-based MREs (MRE-S). The field-dependent rheological properties were studied. Figure 5 shows the loss factor according to the frequency of both MRE-P and MRE-S. In all samples, the loss factor tends to increase with an increased frequency, whereas the loss factor of the MRE-P sample is lower than that of MRE-S. Since the primary factor of the loss factor is related to the interface interaction between the particle and the elastomer, the following results indicate that the relative motion between the particles is increased due to the weak interface bonding between the particles and the medium, which results in energy dissipation. The stronger the interfacial interaction between particles and the medium, the smaller the degree of loss factor. As a result, it confirmed that plate-like CI has stronger interfacial bonds with matrix than spherical CI particles.

### 3.3. Magnetic Property

**Figure 6:**
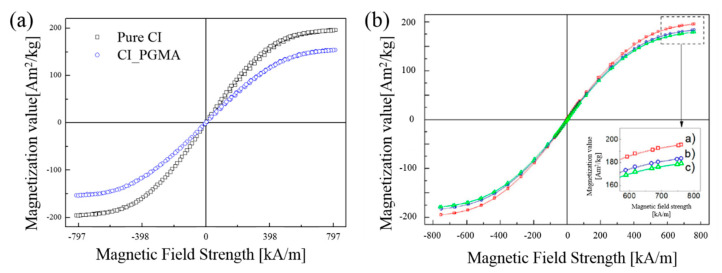
(**a**) VSM curves of pure CI (open dark square) particles and PGMA coated CI (open blue circle) particles [60] and (**b**) magnetization curves of a—bare CI, b—CI-g-PHEMATMS-1 (11 nm), and c—CI-g-PHEMATMS-2 (22 nm) particles obtained via VSM [118].

The magnetic characteristics of the CI particles of the MREs play an important role in their MR properties [116,117]. Knowing the magnetization information of the magnetic particles provides important information for applying the magnetic particles to the MRE. This magnetization investigation is obtained in the form of hysteresis loops as a graph of magnetization value versus magnetic field strength. Figure 6a shows the magnetization curves of pristine CI particles and particles covered with PGMA on the CI surface [60]. Since CI particles are soft magnetics, they show narrow magnetic hysteresis loops. For pure CI, saturation magnetization was 198 Am^2^/kg and CI/PGMA was 153 Am^2^/kg, respectively, confirming that the saturation magnetization is reduced by the coating of PGMA. Figure 6b is the magnetization curve of CI particles coated with poly(trimethylsilyloxyethyl methacrylate) (PHEMATMS) [118]. It indicated that the saturation magnetization value decreased as the PHEMATMS coating thickness increased from 11 nm to 22 nm.

### 3.4. Rheological Characteristics

#### 3.4.1. Dynamic Viscoelastic Property

Both amplitude and frequency sweep tests are being conducted to observe the dynamic mechanical behavior of MREs. MREs are viscoelastic materials that generate and store energy during the deformation process. In general, the ability to store energy is associated to the G′, representing the elastic properties of the material. G” shows the amount of energy dissipation during the deformation process [119]. These G’ and G″ are important parameters for representing viscoelastic properties such as MRE.

##### Amplitude Sweep Test

**Figure 7:**
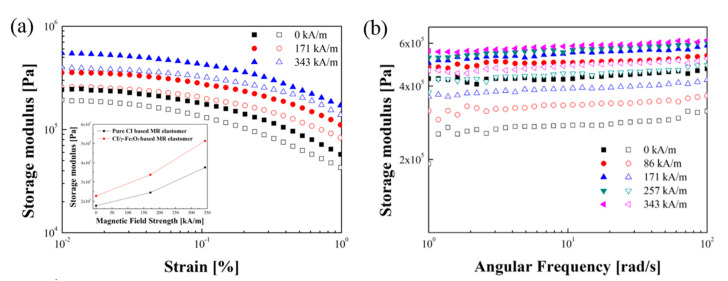
(**a**) Storage Modulus versus strain amplitude sweep and (**b**) storage modulus versus angular frequency sweep (Closed symbol:CI/γ-Fe_2_O_3_ based MRE, open symbol: Pure CI based MRE) [121].

The strain amplitude sweep test examines effect of shear strain on the dynamic moduli of the MRE sample [120]. This test allows to define a linear viscoelastic (LVE) region of the MRE. Figure 7a shows the G’ of the MRE sample as a function of strain amplitude [121]. The strain range is measured from 0.01% to 1% and is measured at 3 different magnetic field intensities. As shown in the figure, the low strain region has a plateau region where the G’, called LVE regime, remains constant. As the strain increases, the G’ tends to decrease gradually, and as the strength of the magnetic field increases, the G’ rises.

##### Frequency Sweep Test

Figure 7b is a frequency sweep test graph [121] of both G’ and G” displayed according to frequency. The constant strain was determined as 0.02% in the LVE region of the strain amplitude sweep test. The measured data range is 1~100 rad/s. As the magnetic field strength increases, the dynamic modulus of the sample increases. At the same magnetic field strength, the G’ of CI/γ-Fe_2_O_3_ based MRE is higher than that of pure CI based MRE as in the amplitude sweep test.

##### MR Effect

MR effect is one of the key parameters for characterizing the performance of MREs. The change in the behaviors of the MR composite under the magnetic field is expressed by the absolute MR effect and the relative MR effect [122,123,124,125]. The absolute MR effect represents the difference between the shear modulus of the maximum value obtained under the magnetic field and the shear modulus (zero field modulus) when there is no magnetic field as given in Equation (2),

(2) $$\Delta G=G_{max}-G_{0}$$

On the other hand, the relative MR effect is the percentage of absolute MR effect and zero field modulus $G_{0}$ as follows;

(3) $$\Delta G_{r}=\frac{\Delta G}{G_{0}}\times 100\%$$

**Figure 8:**
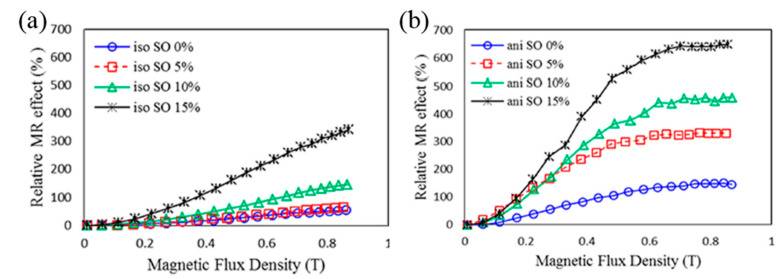
Relative MR effect for (**a**) isotropic MRE and (**b**) anisotropic MRE as a function of magnetic field for different SO contents [97].

As well-known in many studies, the absolute and relative MR effects vary with several factors, including oscillation frequency, magnetic field strength, magnetic particle content, particle size, magnetic material, and the MRE matrix. Khairi et al. [97] studied the effects of silicone oil plasticizer on MREs. Figure 8 shows the relative MR effects of isotropic and anisotropic MREs depending on the amount of silicone oil. Both isotropic and anisotropic MREs show increased relative MR effects as silicone oil content increases. The reason is that the maximum value of shear modulus is similar even though the contents of silicone oil are increased, but as the content of silicone oil is increased, the zero-field modulus (G_0_) of MRE is lowered, which causes a sharp increase in relative MR effect. As such, the MR effect is considered to be a key parameter in evaluating the performance of the MRE.

#### 3.4.2. Creep and Recovery Characteristics

Creep test is an experiment to observe the change of strain with time when certain stress is applied to a sample. In other words, it is a test about the time-dependent mechanical behavior of a sample. Recovery behavior is defined as the change in strain over time that occurs when a material’s stress is abruptly removed. These creep and recovery experiments can be used to infer changes in the microstructure of viscoelastic materials. This helps to understand the properties of the material and the rheological properties of the material. These creep and recovery characteristics play an important role in some engineering applications. In addition, the study of creep and recovery behavior in MREs is very helpful in understanding the deformation mechanism of MR elastomers [126,127,128,129,130].

**Figure 9:**
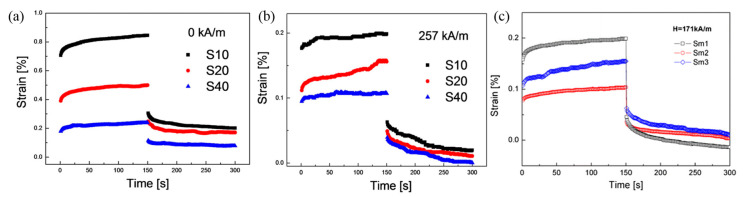
Creep and recovery curves of MREs without (**a**) and with (**b**) the magnetic field (H = 257 kA/m) [131] (**c**) creep and recovery curves of pure elastomer and the MRE samples with the stimuli of magnetic field [126].

Bica et al. [131] prepared MREs by varying the volume fraction of the magnetic particles of the PU base to 10%, 20%, and 40%, and then creep and recovery experiments were conducted. As shown in Figure 9, as soon as the constant stress of 30 Pa is induced, the change of strain occurs immediately. In the case of the sample without a magnetic field, as the amount of CI particles increases, the response strain of the sample decreases, and when the magnetic field is input, the MRE without the magnetic field is measured. This shows that an external magnetic field can cause large stiffness of the MRE. Furthermore, Bica et al. [126] fabricated MREs based on silicone rubber with different CI contents. In this study, as a new application of the MR elastomer, they draw their interest in the achievement of electric capacitors whose capacity, in addition to MR responses, is controlled by an outside magnetic field, along with its preparation and viscoelastic characteristics. They measured creep and recovery curves at constant magnetic field (171 kA/m) and under same stress (30 Pa). As a result, as the content of magnetic particles increased, the recovery ratio decreased.

## 4. Applications of MREs

The studies of the controlling both damping and stiffness of MRE has attracted enormous interests in recent years. The field dependence and controllable of mechanical performance of MRE allows to suit for wide engineering applications as follows.

### 4.1. Vibration Absorber

The various conventional dynamic vibration absorbers (DVAs) are adopted to lower the forced vibration excited at a specific frequency. However, one of drawback of DVAs is the lacking adaptability due to narrow frequency bandwidth. Number of development have been conducted to overcome the disadvantages such as controlling the pressure of air springs [132], altering effective coil number of spring [133], changing the length of threaded flexible rods [134], and minimizing the space between two spring leaves [135]. It is viable to adjust the frequency, the challenges are heavy weight, slow adjusting speed, large dimensions and high energy consumption. Hence one of the high arising solution is MRE. Ginder et al. [136] conducted pioneering work on development on actively controllable MRE vibration absorber. Deng et al. [137] designed series of MRE absorbers working in shear mode, which are able to tune the frequency from 55 to 82 Hz. In addition, Hoang et al. [138,139] fabricated a conceptual ATVA with MRE to minimize vibration inherent for vehicle power train systems.

### 4.2. Vibration Isolator

**Figure 10:**
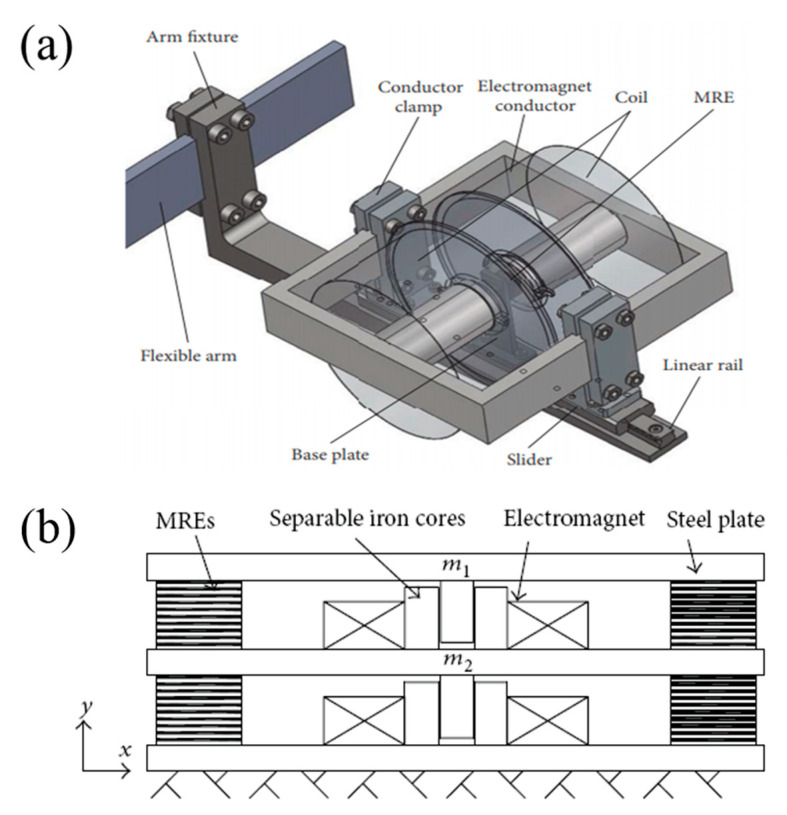
Schematic diagram of (**a**) MRE vibration absorber (Reprinted with permission from [145] and (**b**) MRE isolation system [146].

The smart MRE isolation system, whose stiffness and damping characteristics differ according to the external environment, has been implanted in several studies to overpower the drawbacks of conventional rubber isolators. Many studies have been researched on MRE isolators. Opie et al. [140] researched room temperature vulcanized SR to produce a vertically tunable isolator, which increased the resonance peak attenuation and load speed up to 16% and 30%, respectively. Liao et al. [141] fabricated MRE isolator with a voice coil motor to reduce the transfer rate up to 70 %. Li et al. [142,143] reported a lateral laminated MRE bearing for large scale building isolation, which includes multilayer thin MRE sheets attached on to multilayer thin steel plates. The maximum lateral stiffness that can be changed due to different excitation currents is up to 1630%. In addition, Xing et al. [144], similar bearing structure to Li et al. [143], but for seismic mitigation in a bridge superstructure. The resonance frequency has increased from 10 Hz to 20 Hz, which applies to seismic mitigation. The four MRE isolator embedded together, which is used for the bridge monitoring equipment is given in Figure 10b.

### 4.3. Magnetoresistor Sensor

It is well-known that electrical conductivity of MREs can be affected by the intensity of the applied magnetic field allowing them to be used in various applications [94]. However, MRE is not categorized as electric conductors. MRE is capable of operating as active devices of the electric circuit that allows to control output voltage in magnetic field by addition of iron and graphite microparticles. Therefore, one of the applications of MRE could be a magnetoresistor sensor device. Bica [147] reported that MRE is applicable for quadrupolar magnetoresistor and active element of electric circuit. The preparation of electroconductive MRE was conducted by polymerization of SR with addition of silicone oil, CI microparticles, graphite powder, and catalyst. The electrical conductivity of MRE was found to be affected by magnetic field intensity. Thereby, the voltage at the output terminals of the MR sensor device is considerably influenced by the intensity of the transverse magnetic field, for control voltages kept constant. Hence, electrical conductivity is influenced by magnetic field intensity, applied voltage and time [148].

### 4.4. Electromagnetic Wave Absorber

MREs have been attempted not only in vibration isolators or absorbers but also applicable to electromagnetic wave absorption very recently [149]. The F-Fe/MRE composite was prepared by compression molding and vulcanization using flaky CI particles and SR matrix. The 28 wt.% F-Fe/MRE with 4.3 mm thickness, shows the optimal reflection loss value of −53.3 dB at 4.8 GHz and effective frequency bandwidth (RL < −10 dB) of 6.0 GHz. The combination of magnetic loss, dielectric loss and impedance results matches the multiple reflections and scatterings [149]. In addition, the hybrid MRE is fabricated using silicone oil, rubber, CI particle, graphene nanoparticles and cotton fabric. The dielectric constant and electrical conductivity of hybrid MRE was observed to be enhanced according to the external magnetic field due to relaxation polarization of the SR and interfacial polarization of the graphene [150]. For a potential application on radio-absorbing materials and piezoresistive sensors, Moucka et al. [151] examined relationship between the dielectric response of MREs and their filler clusters’ morphology. Regarding the orientation effect of CI particles, it was further reported that the orientation of CI particles strongly enhances the permittivity of the systems, while preserving their permeability, which ultimately manifests itself in enhanced absorption of electromagnetic energy and reduced thickness of radio-absorbers. Thus, radio-absorbers based on anisotropic MREs are characterized by superior EM shielding capability in the microwave frequency range compared to their isotropic analogues [152].

### 4.5. Other Applications

MRE can be adopted in many other applications. According to Hu et al. [31], the silicone elastomer that consists hard magnetic neodymium-iron-boron (NdFeB) microparticles of 5 µm size was able to act as magneto-elastic soft milimetre-scale robot. This soft robot can move around on surface of liquid, walk and roll on solid surfaces, and crawl in narrow tunnels. The fabrication of small-scale continuum robots with materials of silicone and magnetic particle (NdFeB) enables to navigate through constrained and complex environment for medical applications [153]. In addition, the origami-inspired rapid prototype robot has been fabricated for self-folding, reconfigurable shape, and controllable motility with poly (ethylene glycol) diacrylate and magnetite particles [154].

MRE is also viable for micro-fluid actuation applications. The manipulation of micro-fluid is crucial in biological analysis, optics, chemical synthesis, and information technology. The micro-fluid actuation was achieved using artificial cilia and iron particle with polymer matrix [155]. Another actuated artificial cilia was made using polybutylacrylate and paramagnetic magnetite nano particles to simulate the cilia motion [156]. The MRE has also been used in biomedical application in such a way as active scaffolds for drug and cell delivery. The new active porous scaffold that can be controlled by a magnetic field to transfer different biological agents using alginate hydrogel and magnetite particles [157]. The formation of biofilm on medical device causes serious infections. Hence the engineered tunable active surface topographies with micron-sized pillars was developed using magnetite particles, which was controlled by electromagnetic field [158]. In addition, the MRE could be also applicable in shape-programmable magnetic soft matter, generating new time-varying shapes of soft matter with magnetization profile and actuating field, using ferromagnetic and aluminum particles [159].

Finally, MRE can be used in acoustic metamaterials. The transformation of 3D phase of magnetoactive lattice structures from 2D phase by magnetic field allows to switching signs of constitutive parameters repeatedly. It opens new potential of MRE in acoustic transportation, imaging and refraction [160].

## 5. Conclusions

In this review, we briefly overviewed fabrication, characterizations, properties, and applications of smart MREs composed of various magnetic particles, elastomers, and additives based on the recent studies. Especially, various polymeric elastomeric materials including waste tire rubber are extensively covered. While the morphology of iso/anisotropic MREs was typically observed with SEM images along with their synthesis and physical characteristics, the mechanical properties of MREs are explained based on tensile strength, Payne effect and loss factor. The MR performance of anisotropic MRE was superior than that of isotropic MRE. Accordingly, the mechanical characteristics of surface coated based MRE was observed stronger than the pure CI particle coated MRE due to the enhanced bonding energy between rubber matrix and CI particle. The MR analysis of MRE under various range of magnetic field is conducted with strain amplitude and frequency sweep. According to the MR property measurements, the G’ increases with high magnetic field and at various strain. Hence, the MRE has been drawn a great attention on various applications, such as absorber, isolator, sensor and EMI shielding.

## Figures and Tables

**Table 1:** Mechanical characteristics of anisotropic polyurethane (PU) MREs with different CI concentrations [79].

Iron Contents (%)	Tensile Strength/MPa		Elongation at Break/%		Stress at 100% Strain/MPa		Stress at 200% Strain/MPa		Stress at 300% Strain/MPa	
	Raw	Treated	Raw	Treated	Raw	Treated	Raw	Treated	Raw	Treated
0	9.54		466		1.78		2.33		3.26	
50	9.58	12.07	984	971	1.86	1.92	2.03	2.18	2.32	2.65
60	6.93	7.63	1129	1152	1.66	1.79	1.80	1.97	2.04	2.24
70	1.89	2.34	327	417	1.92	2.23	2.00	2.30	1.99	2.27
